# Sex Dependent Dysregulation of Hepatic Glucose Production in Lean Type 2 Diabetic Rats

**DOI:** 10.3389/fendo.2019.00538

**Published:** 2019-08-06

**Authors:** Chellakkan S. Blesson, Amy Schutt, Shaji Chacko, Juan C. Marini, Pretty Rose Mathew, Daren Tanchico, Meena Balakrishnan, Chandra Yallampalli

**Affiliations:** ^1^Division for Reproductive Endocrinology and Infertility, Department of Obstetrics and Gynecology, Baylor College of Medicine and Family Fertility Center, Texas Childrens' Hospital, Houston, TX, United States; ^2^Department of Pediatrics, Baylor College of Medicine, Children's Nutritional Research Center, Houston, TX, United States; ^3^Critical Care Medicine, Baylor College of Medicine, Houston, TX, United States; ^4^Basic Sciences Perinatology Research Laboratories, Department of Obstetrics and Gynecology, Houston, TX, United States

**Keywords:** developmental programing, glucose intolerance, insulin resistance, glucose production, gluconeogenesis, glycogenolysis

## Abstract

We have characterized a lean type 2 diabetic rat model by gestational low protein programming. We aimed to identify if the regulation of hepatic glucose production (HGP) via gluconeogenesis and glycogenolysis is affected and if there are any sex differences. Fasting (6–7 months old) type 2 diabetic rats received ^2^H_2_O followed by a primed constant rate infusion of [6,6-^2^H_2_] glucose. Blood samples were drawn during steady states after 4 h of fasting and following a euglycemic hyperinsulinemic clamp. HGP and the fraction of glucose derived from gluconeogenesis under fasting and euglycemic states were measured from steady state glucose enrichments after the infusion of [6,6-^2^H_2_]glucose and ^2^H_2_O tracers. Glycogenolysis was determined by calculating the difference between total HGP and gluconeogenesis rates. Hepatic gene expression of enzymes involved in HGP were quantified using qPCR. HGP rates was similar during fasting in both groups and sexes. However, under simulated fed condition, HGP rate was suppressed in controls but not in type 2 diabetic rats. They also showed inefficient HGP suppression in a simulated fed state. Differential analysis showed that suppression of both gluconeogenesis and glycogenolysis under simulated fed state was affected in these low protein programmed type 2 diabetic rats. These effects were greater in females when compared to males. Further, key genes involved in these processes like G6Pase, Pepck, pyruvate carboxylase, and glycogen phosphorylase in liver were dysregulated. Our data shows impaired suppression of HGP via gluconeogenesis and glycogenolysis in type 2 diabetic rats with greater effects on females.

## Introduction

Liver plays a vital role in maintaining glucose homeostasis by regulating glucose production via gluconeogenesis (GNG) and glycogenolysis (GYG) (1). Insulin and glucose levels play vital roles in regulating hepatic glucose production (HGP) by both GNG and GYG (2–5). The insulin control over glucose production is impaired in type 2 diabetes (T2D) (6–8) leading to excess glucose production (8–10). Diabetic patients have increased rates of GNG and impaired suppression of GYG contributing to excessive glucose production leading to elevated blood glucose levels (9). We recently developed a gestational low protein (LP) programmed T2D rat model that closely mimicks human T2D.

Although there are studies on the relationship between glucose production and T2D using rodents, most of them were done on males (11–13) or in prepubertal females (14). There are sporadic reports on the role of glucose production in gestational low LP programmed T2D rat models. T2D rats expressed low LXRα and high 11β-HSD1 and G6Pase (15), and upregulated fetal and maternal hepatic G6Pase and PEPCK activities (16), supporting a role for increased GNG. Another study suggested sex differences in the expressions of PEPCK and 11β-HSD1 in fetal liver when LP was given during the pre-implantation period (17).

Sex steroids testosterone (T) and estradiol (E2) are well-known modulators of insulin sensitivity (18–22). In men, low T causes glucose intolerance and insulin resistance, and T supplementation restored glucose tolerance and insulin sensitivity (21, 22). Lower E2 in women is implicated in T2D (23) and E2 supplementation improved their insulin sensitivity (24). Lower E2 has been shown to cause GI and IR in humans and animal models (25). Further, men with aromatase deficiency (26) and male knockouts for ERα (27) and aromatase (28) in mouse models manifest IR. Thus, it is likely that the modulatory role of sex steroids are responsible for the sex differences in T2D.

Our novel lean T2D rat model develops progressively worsening glucose intolerance and insulin resistance in both sexes with females showing a faster progression and a severe phenotype than males (29). They also have insulin signaling defects in skeletal muscles with clear sex differences (30–32). They developed glucose intolerance, insulin resistance and dysregulated insulin signaling as seen in human T2D (29, 33, 34). In this study we hypothesized that LP programmed T2D rats may have sex dependent dysregulation of HGP rates via both GNG and GYG.

## Materials and Methods

### Animals

The lean diabetic rat model was developed as reported earlier (29). Briefly, pregnant (Day 4) Wistar rats weighing ~230 grams were bought from Harlan Sprague Dawley, Indianapolis, IN. Rats were housed in a temperature-controlled room (23°C) with a 10:14-h light/dark cycle and were given unlimited access to food and water. Pregnant rats were fed with control (20% protein, *n* = 6) or isocaloric low protein (6%, *n* = 6) diet (Harlan Teklad, Madison, WI USA) from day 4 of pregnancy until delivery. Standard laboratory rat chow (Teklad Global 2019, Teklad Diets, Madison WI) was given to the dams after delivery until the end of weaning and pups were given the standard laboratory rat chow after weaning. To ensure equal access to nutrition, pups with extreme weights were culled on day 1 after birth to normalize the litter size to 8 pups per mother. Both male and female pups (6–7 months old) were used for all the glucose production studies. All procedures (including euthanasia) were performed during diestrus phase in females. Tissues were collected, snap frozen in liquid nitrogen and stored at −80°C until analysis. The experimental animal numbers were based on the minimal number of animals required to obtain significance at the *p* < 0.05 confidence level. For *n* = 6/group was used based on a power analysis preformed with estimates of variation drawn from preliminary data, a power of 0.8 and a *p* ≤ 0.01. All experimental procedures involving rats were approved by the Institutional Animal Care and Use Committee of the Baylor College of Medicine, Houston, Texas.

### Glucose Production and Euglycemic Hyperinsulinemic Clamp

**Figure 1:**
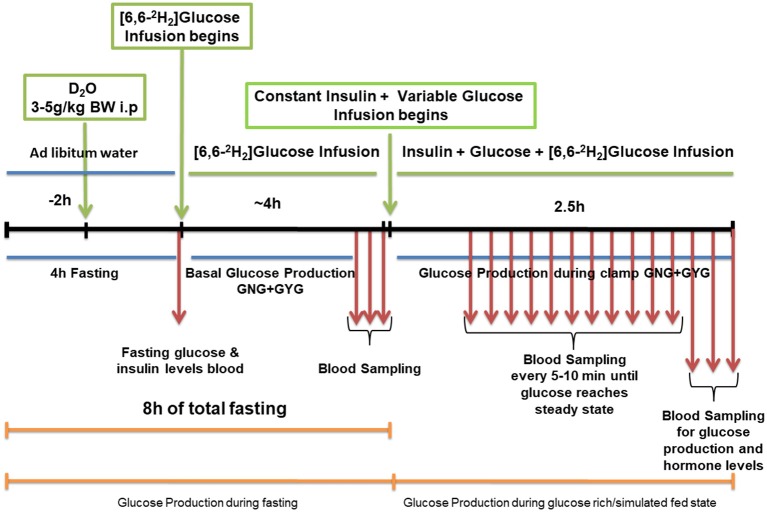
Schematic work flow for measuring hepatic glucose production in low protein diet programmed lean type 2 diabetic (LP) rats during fasting and simulated fed condition (Image not to scale).

Isotopic infusion and sampling were done using tail vein catheters. Rats were restrained and tail vein catheters were inserted as described previously (35). BD Insyte™ Autoguard™ catheters were used for both infusion and sampling. The infusion catheter was placed on one side of the lateral tail vein proximal to the body and the sampling catheter was placed distally on the contralateral side. The catheters were connected by tubing to syringe pumps. Infusion tubing had a three-way union with valves connecting to glucose on a separate pump for euglycemic hyper insulinemic clamp. Infusions were performed using The PHD ULTRA™ syringe pumps (Harvard Apparatus Inc., Cambridge MA). Rate of appearance of glucose and GNG was measured at the end of 8 h of fasting. Rats received a dose of ^2^H_2_O (4 mg/g BW) resulting in a deuterium enrichment of ~0.5% in body water. Two hours after the ^2^H_2_O dose, a constant infusion of [6,6-^2^H_2_]glucose at 0.75 mg^*^kg^−1*^min^−1^ (200 μL/hour for males and 150 μL/hour for females) was started and continued for 4 h. A baseline blood sample was taken prior to the infusion. After the isotopic infusion, blood was drawn from the sampling catheter or tail tip three times at 5 min intervals (fasting sample). This was immediately followed by an euglycemic hyperinsulinemic clamp (2 h and 30 min). When the blood glucose concentration reached a steady state after glucose infusion, three blood samples were taken (simulated fed/glucose rich condition). Blood samples were processed for isotopic analysis. A schematic diagram of the schedule is as shown in Figure 1. The isotopic enrichment of [6,6-^2^H_2_]glucose was measured by gas chromatography-mass spectrometry (GCMS) using the pentaacetate derivative (36–39). Incorporation of deuterium in glucose from ^2^H_2_O was determined by the average deuterium enrichment in glucose carbons 1,3,4,5, and 6 (37, 39, 40). Deuterium enrichment in plasma water was determined by Isotope Ratio Mass Spectrometry (IRMS). These analyses provided both total glucose production rates and absolute rates of GNG under fasting and simulated fed states. The rate of GYG was calculated by the difference between total glucose production rate and GNG rate as reported earlier (39).

### Gene Expression

Hepatic gene expression of key enzymes involved in GNG and GYG was quantified using qPCR. RNA was isolated TRIzol reagent (Life Technologies, Carlsbad, CA) and further purified with RNeasy clean-up kit (Qiagen, Valencia, CA). All RNA samples were treated with DNase. RNA concentration and purity were determined using an ND-1000 model Nanodrop spectrophotometer (Thermo Fisher Scientific, Newark, DE). Two micrograms of total RNA were reverse transcribed (RT) using a modified Maloney murine leukemia virus-derived RT (New England Biolabs Inc., MA, USA) and random hexamer primers (Life Technologies, CA, USA) as reported earlier. cDNA was amplified by real-time PCR using SYBR Green (Bio-Rad, Hercules, CA) in a CFX96 model real-time thermal cycler (Bio-Rad). Specific pairs of primers (IDT®, IA, USA) (Table 1), were used for each gene amplification. PCR conditions used were 10 min at 95°C for 1 cycle, 15 s at 95°C, 30 s at 60°C, and 15 s at 72°C for 40 cycles, followed by a melt curve analysis (0.5°C/ 5 s from 65 to 95°C). Results were calculated using 2^−ΔΔ*CT*^ method and expressed as fold changes of expression of genes of interest. All reactions were performed in duplicates with Cyclophilin A as internal control with *n* = 5–6 per group.

**Table 1:** Oligonucleotide primers used for real-time PCR.

**Genes**	**Accession No**.	**Primers *F* = forward; *R* = reverse**
Pyruvate carboxylase	NM_012744.2	F: 5′-GCTGCGGCAGGAAAACATC-3′ R: 5′-CACCACTCCGGAAAACCTCA-3′
Pepck	NM_198780.3	F: 5′-TGCCCATCGAAGGCATCATT-3′ R: 5′-GGTGGCCTCTGATCTCATGG-3′
G6pase	NM_013098.2	F: 5′-TGAGACTGGACCAGGGAGTC-3′ R: 5′-AGCACCGGAATCCATACGTT-3′
Glycogen phosphorylase (liver)	NM_022268.1	F: 5′-ATGACAAGTGCCCCAAGAGG-3′ R: 5′-AGCCCGAGCTGGTAAATAGC-3′
Glycogen phosphorylase (muscle)	NM_012638.1	F: 5′-GTTTCCTTAATCGGGTGGCG-3′ R: 5′-GTGTGCCATGTTGATGCGTT-3′
Hexokinase 1	NM_012734.1	F: 5′-GCTCACGAGGGGAAAGTTCA-3′ R: 5′- CAACATCAGACGGCTCCACT-3′
Cyclophilin A	NM_017101.1	F: 5′-TATCTGCACTGCCAAGACTGACTG-3′ R: 5′-CTTCTTGCTGGTCTTGCCATTCC-3′

### Western Blots

Western blots for hepatic enzymes were performed as reported earlier (32). Briefly, 10–30 μg of proten extract was resolved on 4–15% precast gradient polyacrylamide gels (Mini-PROTEAN TGX Precast Gels; Bio-Rad, Hercules, CA). Resolved proteins were transferred to a polyvinylidine fluoride membrane (Millipore, Billerica, MA). Primary antibodies were incubated overnight at 4°C after blocking the membranes in 5% bovine serum albumin or nonfat dried milk in Tris buffered saline containing 0.1% Tween 20 for 1 hour at room temperature. Details of primary antibodies and their dilutions are as follows: Pyruvate carboxylase (Cat # ab126707,1:1000), PEPCK (Cat # ab 70358, 1:1000) and G6Pase (Cat # ab 83690, 1:1000) were obtained from Abcam, Cambridge, MA USA. Antibodies for hexokinase II (Cat # 2867, 1:1000) and GAPDH (Cat # 60004-1-Ig, 1:5000) were obtained from cell signaling, Danvers, MA, USA and Proteintech inc. Rosemont, IL USA respectively. After primary antibody incubations, membranes were washed and incubated for 60 min at room temperature with horseradish peroxidase conjugated secondary antibodies (Abcam Cambridge, MA USA). Membranes were washed and incubated in ECL Western blotting detection reagents (Pierce Biotechnology, Waltham, MA USA) for a minute and imaged using the Odyssey Fc imaging system (LI-COR). Densitometric analyses were performed using Image Studio software from LI-COR.

### Statistical Analyses

Statistical analyses were performed using GraphPad Prism software. Data are presented as Mean ± SEM. Comparison between two groups was performed using unpaired student's *t*-test. When comparisons between multiple groups with two factors were done, statistics was performed with two-way ANOVA followed by Bonferroni test. Differences were considered significant when *p* < 0.05.

## Results

### Total Hepatic Glucose Production Rate in Males

**Figure 2:**
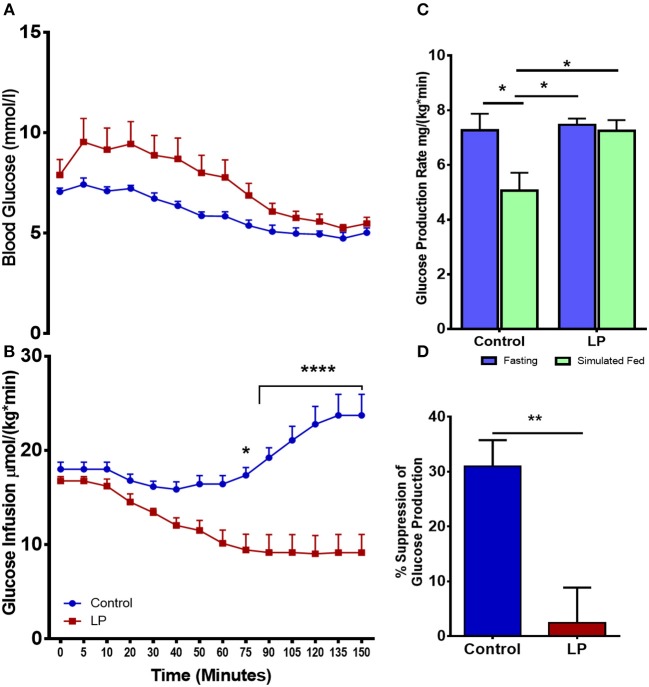
Impaired insulin induced suppression of glucose production during fasting and simulated fed state from 6 month old male lean type 2 diabetic (LP) rats. **(A,B)** Euglycemic hyperinsulinemic clamp for low protein diet programmed lean type 2 diabetic (LP) males in comparison with controls along with **(C)** total hepatic glucose production rate during fasting and simulated fed state. **(D)** Percentage suppression of glucose production in males during simulated fed state. ^*^p < 0.05, ^**^p < 0.01, and ^****^p < 0.0001, *n* = 5–6.

Total hepatic GPR was measured after 8 h of fasting and under simulated fed condition by glucose infusion (Figures 2A–D). Data from the euglycemic hyperinsulinemic clamp demonstrated that T2D males are markedly insulin resistant compared to controls, as the glucose infusion rate during euglycemia was nearly 2.5-fold lower in T2D males when compared to their controls (Figures 2A,B). We further show that the rate of total glucose production was 7.3 ± 0.6 mg^*^kg^−1*^min^−1^ in fasting controls. However, upon glucose infusion the total glucose production rate decreased to 5.1 ± 0.7 mg^*^kg^−1*^min^−1^ (*p* < 0.05) (Figure 2C) showing 31 ± 4% suppression of glucose production (Figure 2D). Interestingly, in the LP programmed T2D rats, the rate of total glucose production was 7.5 ± 0.2 mg^*^kg^−1*^min^−1^ during fasting and 7.2 ± 0.4 mg^*^kg^−1*^min^−1^ upon glucose infusion (Figure 2C) showing only ~2% ± 6 suppression of glucose production (Figure 2D). These results indicate compromised glucose suppression in T2D males as the percentage suppression of glucose production was significantly lower in T2D compared to control males (Figure 2D).

### Total Hepatic Glucose Production Rate in Females

**Figure 3:**
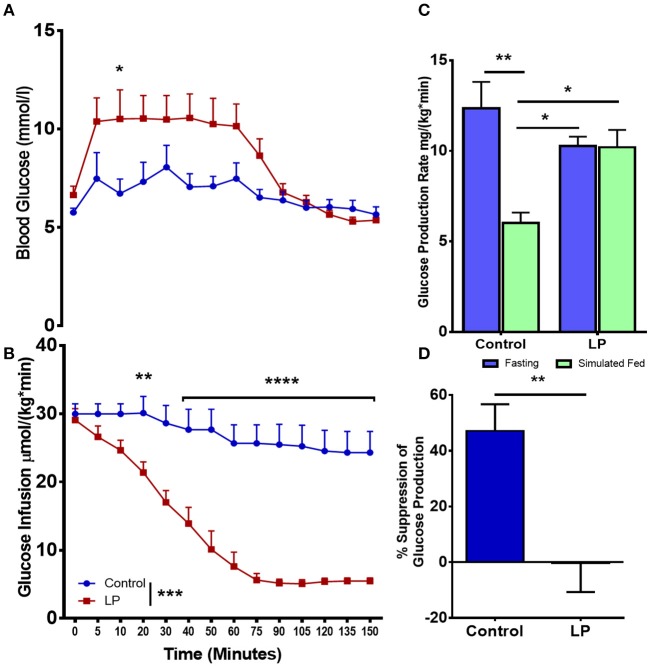
Impaired insulin induced suppression of glucose production during fasting and simulated fed state from 6 month old low protein programmed lean type 2 diabetic (LP) female rats. **(A,B)** Euglycemic hyperinsulinemic clamp for type 2 diabetic (LP) females in comparison with controls. **(C)** Total hepatic glucose production rate during fasting and simulated fed state. **(D)** Percentage suppression of glucose production in males during simulated fed state. *p < 0.05, ^**^p < 0.01, ^***^p < 0.001, and ^****^p < 0.0001, *n* = 5–6.

In female rats (Figures 3 A–D), our euglycemic hyperinsulinemic clamp results showed that during euglycemia, the glucose infusion rate was nearly 5-fold lower in T2D females when compared to their respective controls indicating that LP programmed T2D females were insulin resistant (Figures 3A,B). The rate of total glucose production in the control group was 12.4 ± 1.5 mg^*^kg^−1*^min^−1^ during fasting and the glucose production decreased by 47 ± 8 to 6.0 ± 0.6 mg^*^kg^−1*^min^−1^ (*p* < 0.01) during glucose infusion (Figure 3C). In the T2D group, the rate of total glucose production was 10.3 ± 0.5 mg^*^kg^−1*^min^−1^ during fasting. Upon glucose infusion, the rate of glucose production (10.2 ± 1.0 mg^*^kg^−1*^min^−1^) did not change significantly from the rate of glucose production during fasting (Figure 3C). These results indicate that there was no suppression of the HGP rate in LP induced T2D females indicating a complete breakdown of the regulation of glucose production (Figure 3D).

### Gluconeogenesis and Glycogenolysis in Males

**Figure 4:**
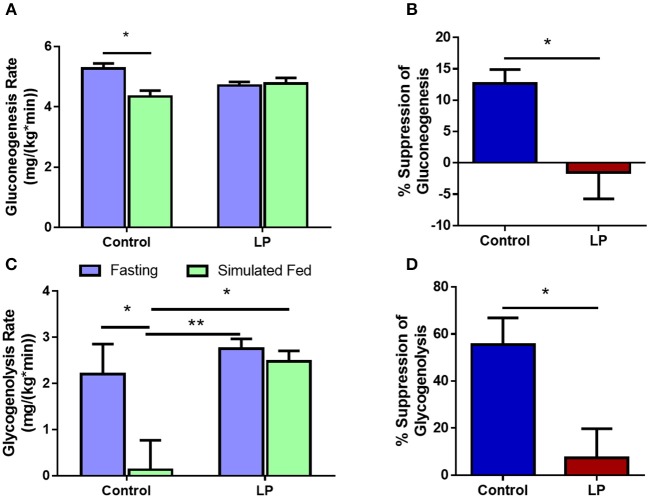
Hepatic glucose production in males via gluconeogenesis **(A,B)** and glycogenolysis **(C,D)** by control and low protein programmed lean type 2 diabetic (LP) rats during fasting and simulated fed states. ^*^p < 0.05 and ^**^p < 0.01, *n* = 5–6.

Glucose production via hepatic GNG and GYG was measured after 8 h of fasting and under simulated fed condition by glucose infusion. In males, our data showed that the rate of glucose production via GNG was 5.3 ± 0.2 mg^*^kg^−1*^min^−1^ in controls during fasting, decreasing to 4.3 ± 0.2 mg^*^kg^−1*^min^−1^ (*p* < 0.05) (Figure 4A) under simulated fed condition by glucose infusion showing 13 ± 2% suppression of glucose production by GNG (Figure 4B). However, in T2D males, there were no differences (−2 ± 4%) in the rate of production of glucose by GNG with 4.7 ± 0.1 mg^*^kg^−1*^min-1 during fasting and 4.8 ± 0.2 mg^*^kg^−1*^min^−1^ during glucose infusion (Figure 4A). Furthermore, in controls the rate of GYG was 2.2 ± 0.7 mg^*^kg^−1*^min^−1^ during fasting and the rate of GYG decreased to 0.1 ± 0.6 mg/(kg^*^min) (*p* < 0.05) upon simulated feeding by glucose infusion (Figure 4C). However, in T2D males, the GYG rates were similar during fasting (2.8 ± 0.2 mg^*^kg^−1*^min^−1^ in controls vs. 2.5 ± 0.2 mg^*^kg^−1*^min^−1^ in T2D) and simulated fed condition (Figure 4C). The percentage suppression of glucose production via GYG upon simulated feeding was greater (*P* < 0.05) in controls with 56 ± 9% suppression when compared to 7 ± 11% in T2D group males (Figure 4D).

### Gluconeogenesis and Glycogenolysis in Females

**Figure 5:**
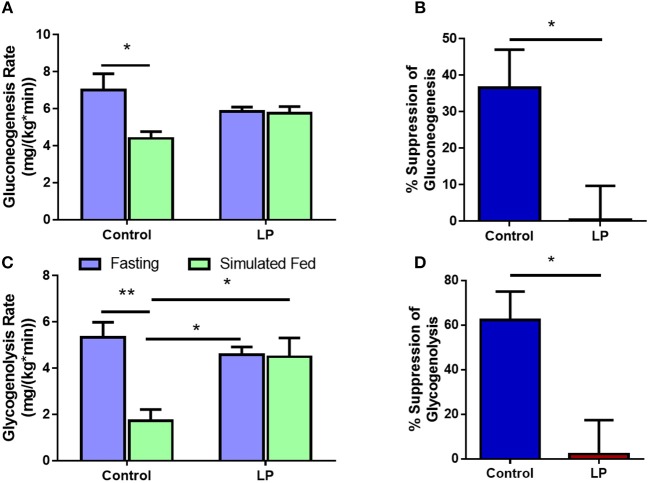
Hepatic glucose production in females via gluconeogenesis **(A,B)** and glycogenolysis **(C,D)** by control and type 2 diabetic (LP) rats during fasting and simulated fed states. *p < 0.05 and **p < 0.01, *n* = 5–6.

In female rats, the rate of glucose production via GNG was 7.0 ± 0.8 mg^*^kg^−1*^min^−1^ during fasting in control group and the rates decreased by 37 ± 8% to 4.4 ± 0.4 mg^*^kg^−1*^min^−1^ (*p* < 0.01) during glucose infusion (Figures 5A,B). In T2D group, the rate of glucose production by GNG was 5.8 ± 0.2 mg^*^kg^−1*^min^−1^ during fasting and upon glucose infusion the rate of glucose production did not show any change and the values were similar to that of fasting at 5.7 ± 0.4 mg^*^kg^−1*^min^−1^ during glucose infusion (Figure 5A). Further, in controls the rate of GYG was 5.3 ± 0.7 mg^*^kg^−1*^min^−1^ during fasting and the rate of production GPR via GYG decreased significantly to 1.7 ± 0.5 mg^*^kg^−1*^min^−1^ (*p* < 0.01), upon simulated feeding by glucose infusion (Figure 5C). However, in T2D females, the rate of glucose production did not show any difference between fasting and simulated fed condition (4.6 ± 0.3 mg^*^kg^−1*^min^−1^ in controls vs. 4.5 ± 0.8 mg^*^kg^−1*^min^−1^ in T2D) (Figure 5C). In females, the percentage suppression of glucose production via GYG upon simulated feeding was greater (*P* < 0.05) in controls with 62 ± 11% suppression when compared to 2 ± 14% in T2D group females (Figure 5D).

### Hepatic Gene mRNA and Protein Expression

**Figure 6:**
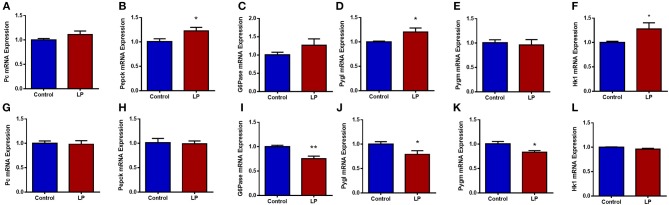
Hepatic expression of genes related to glucose metabolism in control and low protein programmed lean type 2 diabetic (LP) in male **(A–F)** and female **(G–L)** rats. Gene expressions were assessed by qPCR with Cyclophilin A as reference gene and expressed as arbitrary units. *p < 0.05, **p < 0.01, *n* = 5–6.

**Figure 7:**
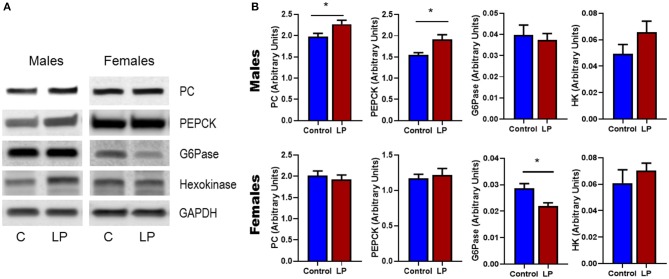
Western Blots **(A)** and its corresponding desitometric analysis **(B)** showing the protein levels of key enzymes related to glucose metabolism in control and low protein programmed lean type 2 diabetic (LP) in male and female rats. *p < 0.05, *n* = 4–6.

Expression of genes related to the regulation of GNG and GYG were quantified in liver. In males, there was an upregulation of glycogen phosphorylase (*P* < 0.05), PEPCK (*P* < 0.05) and hexokinase (*P* < 0.05) in T2D livers when compared to controls (Figures 6 A–F). Pyruvate carboxylase and G6Pase showed an increasing trend but did not reach statistical significance. In T2D females, G6Pase (*P* < 0.01) and glycogen phosphorylase (*P* < 0.05) were downregulated, however other genes such as pyruvate carboxylase, PEPCK and hexokinase did not show any difference when compared to the controls (Figures 6 G–L). Protein levels of key hepatic enzymes reflect mRNA levels (Figures 7 A,B). In males, pyruvate carboxylase and PEPCK were significantly upregulated (*P* < 0.05) and hexokinase showed similar trend but did not reach statistical significance. G6Pase did not show any change between the groups. In females, G6pase was significantly downregulated (*P* < 0.05), other genes did not show any significant changes (Figures 7A,B).

## Discussion

T2D is a major epidemic with 1 in 10 affected in the US and an estimated increase to 1 in 3 by 2050 (41). Most investigations have focused on the obese population; however, there is a distinct sub-population of T2D patients who are lean with normal BMI (33). Although T2D is attributed to lifestyle and genetics, recent studies indicate that adverse *in utero* nutrition could cause T2D later in life and lean T2D has primarily been attributed to *in utero* nutritional imbalances (42, 43). Studies from India (up to 26% of T2D patients worldwide) and Caribbean islands (5% of T2D patients) report that a significant number of T2D patients are lean (34, 44, 45). Further, a study on American minorities showed that 13% of T2D patients are lean (34, 46) with a 5-fold higher incidence in Asians when compared to other ethnicities (46). A recent German study reported that 8.4% of the T2D patients were lean with higher mortality than obese patients (47). These recent studies show the prevalence of lean T2D and its importance to understand the mechanism and pathophysiology. Using our novel lean model, we show for the first time that glucose production by both GNG and GYG are affected in lean T2D rats. We had earlier characterized and showed that this model develops glucose intolerance and insulin resistance (29).

Glucose homeostasis is achieved by the dynamic control of blood glucose levels by various processes involving glucose utilization, storage and production under the influence of various hormones and enzymes (1, 48). Liver plays a major role as it stores glucose in the form of glycogen and is also the dominant organ involved in endogenous glucose production via GNG and GYG (1). Abnormal regulation of glucose causes hypoglycemia or hyperglycemia leading to various deleterious clinical outcomes. Our present study shows that the regulation of total glucose production is compromised as shown in diabetic patients (8, 10, 49). Suppression of total HGP was severely affected in both sexes. Earlier studies in T2D patients have shown that the insulin dependent regulation of glucose production is affected leading to increased glucose production (4, 50) and several studies have shown that GNG may play a dominant role in this process (51, 52). Interestingly, our study shows that there is no net increase in glucose production either by GNG or GYG during fasting in both sexes. However, the suppression of glucose production is impaired with a more prominent dysregulation on GYG than GNG. It is likely that glucose production via GYG is a short-term effect as glycogen storage is limited whereas GNG may have a lasting effect. In a study using obese Zucker rats, it was reported that there was an increase in GYG during fasting (53). However, in our lean T2D model we do not see any such increase; rather, the suppression of GYG is impaired during a simulated fed condition. In T2D patients, reports on the rate of GYG are varied with increase (9), decrease (52) or no change (54). The differences could be attributed to differences in methodology, population characteristics, BMI and/or severity of the disease. Our lean T2D rats have normal fasting insulin and glucose levels but have high insulin and glucose levels after a glucose bolus during GTT and increased insulin resistance during an euglycemic hyperinsulinemic clamp (29). Our data shows that the insulin induced suppression of glucose production is compromised as observed in T2D patients (6). Loss of hepatic insulin signaling could be responsible for hepatic dysfunction causing impaired suppression of glucose production (55).

There are sex differences in the action of insulin in liver and hepatic glucose production (56–58). We had previously showed that females develop glucose intolerance earlier and the disease progresses faster compared to males in this lean T2D model (29). The present study shows that hepatic glucose production and the suppression of glucose production via GYG and GNG are greater in females when compared to males. Consequently, in the T2D group the suppression of glucose production is completely lost upon simulated feeding causing a more severe phenotype in females than males. Sex differences in developmental programming have been reported in different models and in different facets of metabolism (59). To the best of our knowledge, this is the first report on the regulation of HGP via both GYG and GNG in lean T2D. There are previous reports on the sex differences on genes related to GNG (17), our study shows clear evidence of sex differences at the physiological level. Although the mechanisms are not clearly understood, there are reports that developmental programming can cause structural and functional changes in liver (60). LP programmed male offspring have been shown to have altered expressions of PEPCK and glucokinase, further, showed impaired suppression of glucose output despite high expression of insulin receptors (60, 61).

Gene expression data showed dysregulation of key enzymes and could be responsible for the impaired suppression of insulin induced glucose production. Both mRNA and protein levels show similar tendencies in both sexes. Interestingly, males and females had distinctly different dysregulations via different genes. An earlier study also suggested sex differences in the expressions of PEPCK and 11β-HSD1 in fetal liver in a different LP animal model (17). Human studies in T2D have shown sex differences in glucose turnover and hepatic insulin action (56). Although there are a few studies on sex differences in developmental programing and T2D, most studies have been done in males (59). These data clearly show that GYG and GNG are differentially modulated in a sex dependent fashion showing the importance in understanding sex specific disease mechanisms. Sex steroids could play a modulatory role causing sex differences but the exact mechanism of regulation is not clearly understood.

One of the weaknesses of our study is the absence of enzyme activity data of the enzymes involved in GYG and GNG. Enzymology data during fasting and simulated fed states would have given us additional insights into the mechanisms of the disease and if there are any sex differences. However, absolute measurement of total glucose production and GNG using isotope based GCMS has revealed that in lean T2D, insulin control of glucose production is compromised leading to unregulated HGP even under glucose rich state leading to excessive blood glucose levels.

In summary, our study on the lean T2D rat model shows that developmental programming *in utero* by a low protein diet affects HGP via both GNG and GYG. Further, we also show sex differences in the suppression of glucose production upon simulated feeding with greater dysregulation in females when compared to males. The exact mechanism of how developmental programming affects HGP and the reasons for these sex differences remain to be elucidated.

## Ethics Statement

All experimental procedures involving rats were approved by the Institutional Animal Care and Use Committee of the Baylor College of Medicine, Houston, Texas.

## Author Contributions

CB and CY had substantial contributions to conception, design, acquisition, and interpretation of data. CB, CY, AS, JM, and SC contributed to draft the manuscript. CB, AS, JM, PM, DT, and MB contributed to the animal and bench work. SC performed all GC-MS and associated analysis.

### Conflict of Interest Statement

The authors declare that the research was conducted in the absence of any commercial or financial relationships that could be construed as a potential conflict of interest.
